# Short-Term Water- and Land-Based Exercise Training Comparably Improve Exercise Capacity and Vascular Function in Patients After a Recent Coronary Event: A Pilot Randomized Controlled Trial

**DOI:** 10.3389/fphys.2019.00903

**Published:** 2019-07-16

**Authors:** Danijela Vasić, Marko Novaković, Mojca Božič Mijovski, Breda Barbič Žagar, Borut Jug

**Affiliations:** ^1^Faculty of Medicine, University of Ljubljana, Ljubljana, Slovenia; ^2^Terme Krka, Šmarješke Toplice, Slovenia; ^3^Department of Vascular Diseases, University Medical Center, Ljubljana, Slovenia; ^4^Laboratory for Haemostasis and Atherothrombosis, Department of Vascular Diseases, University Medical Center, Ljubljana, Slovenia; ^5^KRKA, d.d., Novo Mesto, Slovenia

**Keywords:** coronary artery disease, cardiac rehabilitation, aquatic exercise, myocardial infarction, exercise training

## Abstract

**Background:**

We hypothesized that a 2-week twice daily aquatic endurance *plus* calisthenics exercise training program: (i) increases aerobic exercise capacity (peak oxygen uptake/$\dot{V}$O_2_peak), (ii) improves endothelium-dependent flow-mediated vasodilation (FMD), and (iii) reduces circulating markers of low-grade inflammation and hemostasis, as compared to land-based endurance *plus* calisthenics exercise training or no exercise in patients undergoing short-term residential cardiac rehabilitation after a recent coronary artery disease (CAD) event.

**Methods:**

Patients with a recent myocardial infarction or revascularization procedure were randomized into two interventional groups and a control group. The interventional groups underwent supervised aerobic endurance *plus* calisthenics exercise training either in thermo-neutral water or on land at moderate intensity (60–80% of the peak heart rate achieved during symptom-limited graded exercise testing) for 30 min twice daily for 2 weeks (i.e., 24 sessions). The control group was deferred from supervised exercise training for the 2-week duration of the intervention, but was advised low-to-moderate intensity physical activity at home while waiting. At baseline and after the intervention period, all participants underwent estimation of aerobic exercise capacity, brachial artery flow-mediated dilatation (FMD, measured ultrasonographically at rest and during reactive hyperemia after 4.5 min of forearm cuff inflation), markers of cardiac dysfunction (NT-proBNP), inflammation (hsCRP, IL-6, IL-8, IL-10), cell adhesion (ICAM, P-selectin), and hemostasis (fibrinogen, D-dimer).

**Results:**

A total of 89 patients (mean age 59.9 ± 8.2 years, 77.5% males, $\dot{V}$O_2_peak at baseline 14.8 ± 3.5 ml kg^-1^ min^-1^) completed the study. Both exercise modalities were safe (no significant adverse events recorded) and associated with a significant improvement in $\dot{V}$O_2_peak as compared to controls: age and baseline $\dot{V}$O_2_peak-adjusted end-of-study $\dot{V}$O_2_peak increased to 16.7 (95% CI 16.0–17.4) ml kg^-1^ min^-1^ with land-based training (*p* < 0.001 for change from baseline) and to 18.6 (95% CI 17.9–19.3) ml kg^-1^ min^-1^ with water-based training (*p* < 0.001 for change from baseline), but not in controls (14.9 ml kg^-1^ min^-1^; 95% CI 14.2–15.6; *p* = 0.775 for change from baseline). FMD also increased in both intervention groups (from 5.5 to 8.8%, *p* < 0.001 with land-based, and from 7.2 to 9.2%, *p* < 0.001 with water-based training, respectively), as compared to controls (*p* for change 0.629). No significant changes were detected in biomarkers of inflammation, cell adhesion or hemostasis, whereas levels of NT-proBNP (marker of cardiac dysfunction) decreased in the water-based training group (*p* = 0.07 vs. controls).

**Conclusion:**

Endurance *plus* calisthenics exercise training in thermo-neutral water is safe, and improves aerobic exercise capacity and vascular function in patients undergoing short-term residential cardiac rehabilitation after a recent CAD event.

**Clinical Trial Registration:**

www.ClinicalTrials.gov, identifier NCT02831829.

## Introduction

Exercise-based cardiac rehabilitation remains a cornerstone of management and secondary prevention in patients with coronary artery disease (CAD) (Piepoli et al., 2010; Anderson et al., 2016). Acute coronary events – such as a recent myocardial infarction and/or coronary artery bypass grafting (CABG) procedure – may impair the ability of individuals to engage in exercise because of cardiac dysfunction, risks associated with the acute effects of exercise, post-procedure recovery, or immediate post-event psychological concerns (Anderson et al., 2016). In this respect, cardiac rehabilitation – either in outpatient settings or as an intensive short-term residential program – provides sufficient monitoring and reassurance to patients in the immediate aftermath of a recent CAD event, thus empowering them to confidently adopt long-term regular exercise and a healthy lifestyle (Mampuya, 2012; Menezes et al., 2014).

Aerobic exercise training on land (such as cycling, walking, jogging, or rowing) – either alone, or supplemented by non-weight bearing exercises (calisthenics) or low-weight resistance training – has been the most studied and therefore the most widely implemented (Bjarnason-Wehrens et al., 2010) exercise modality in cardiac rehabilitation programs. On the one hand, this exercise modality is safe in CAD patients, purportedly because it provides a regulated cardiac output increase to meet the perfusion demand of large exercising muscle groups, thus minimizing safety concerns over raised pre- and after-load in high-risk cardiac patients with low aerobic exercise capacity – such as those after a recent myocardial infarction or revascularization procedure (Balady et al., 2007). On the other hand, this exercise modality has been shown to mitigate risk factors and metabolic abnormalities (Casillas et al., 2007; Ismail et al., 2013), which contribute to CAD and its progression, as well as to improve endothelial dysfunction, which plays a central role in all stages of atherosclerosis. In patients with CAD, endothelium-dependent vascular dysfunction may be associated with impaired systemic (skeletal muscle) and coronary (myocardial) perfusion, resulting in exercise intolerance and myocardial ischemia at exertion, respectively (Bruning and Sturek, 2015). Moreover, endothelial dysfunction in CAD is associated with low-grade inflammation and increased monocyte adhesion, which promote atherosclerotic plaque build-up and rupture, as well as with increased hemostatic activity, which promotes coronary thrombosis (Kokkinos and Myers, 2010; Winzer et al., 2018). Conversely, land-based exercise training has been shown to revert or reduce these abnormalities. In a seminal study by Hambrecht et al. (2000), 4 weeks of in–hospital bicycle ergometer training improved endothelium–dependent arterial vasomotion in patients with CAD, likely through restoring the balance between synthesis and depletion of vasoactive and vasoprotective nitric oxide (NO) (Hambrecht et al., 2003). Mechanistically, this is largely due to a response to exercise-induced shear stress (Kokkinos and Myers, 2010; Bruning and Sturek, 2015). In addition, regular aerobic exercise training on land is associated with a reduction in low-grade inflammation [as assessed by high-sensitive C-reactive protein (hsCRP) levels], cell adhesion (as assessed by cell adhesion molecules, such as ICAM and P-selectin) and hemostatic activity (as assessed by markers of coagulation and fibrinolysis, such as fibrinogen and D-dimer) (Womack et al., 2003; Pedersen, 2017), suggesting an interplay between the effects of aerobic exercise on the endothelium, low-grade inflammation, cell adhesion, and hemostasis in patients with CAD (Kokkinos and Myers, 2010; Winzer et al., 2018).

In contrast to exercise on land, the implementation of aquatic exercises in cardiac rehabilitation programs remains debated (Lazar et al., 2013). Concerns have been traditionally raised over possible adverse cardiovascular effects of exercising in water – namely, an increased preload due to water immersion (i.e., hydrostatically driven raise in venous return and central venous pressure, possibly yielding ventricular dysfunction) (Meyer, 2006; Pendergast et al., 2015; Shah et al., 2017) and/or an increased afterload due to temperatures below thermo-neutrality (i.e., vasoconstriction in cold water associated with a risk of dysrhythmias) (Schmid et al., 2009). On the other hand, the very same hemodynamic effects of thermo-neutral water immersion in patients with CAD have been associated with favorable improvements in cardiac performance and peripheral vascular reactivity after as little as 3 weeks of aquatic cardiac rehabilitation, suggesting pronounced exercise-induced shear stress as a possible mechanism (Mourot et al., 2010). Yet, previous research on the impact of aquatic exercise in patients with CAD is limited when compared with land-based modalities (Schmid et al., 2007, 2009; Volaklis et al., 2007; Laurent et al., 2009; Teffaha et al., 2011; Choi et al., 2015), and marred by small number of participants, selective patient inclusion [limited to stable CAD (Volaklis et al., 2007; Teffaha et al., 2011) or to patients achieving > 7 METs at exercise testing, (Tokmakidis et al., 2008) thus not reflecting the patient populations referred for cardiac rehabilitation in the immediate aftermath of a CAD event], and inferior study design [e.g., pre-post studies without comparator groups (Korzeniowska-Kubacka et al., 2016) or non-randomized patient separation] (Tokmakidis et al., 2008), with four notable exceptions. Volaklis et al., 2007 randomized 24 patients with stable CAD to 16 weeks of either water cycling *plus* water games, land cycling *plus* resistance training, or no exercise at all, and showed that water- and land-based protocols comparably improved exercise test time, muscle strength and blood lipid profiles. Conversely, Lee et al. (2017) randomized 60 older (>65 years old) patients with CAD and osteoarthritis to 24 weeks of either aqua walking or treadmill walking, and showed that both protocols comparably improved aerobic exercise capacity, but the improvements in body composition and lipid levels were significantly more pronounced with aqua walking. Teffaha et al. (2011) randomized 24 patients with CAD and 24 patients with heart failure to 3 weeks of cardiac rehabilitation, comprising land cycling *plus* calisthenics either on land or in water; both protocols were associated with significant increase in aerobic exercise capacity in patients with CAD. Similarly, Laurent et al. (2009) randomized 24 patients with CAD and 24 patients with heart failure to 3 weeks of cardiac rehabilitation, comprising land cycling *plus* gymnastics either on land or in water; both protocols improved aerobic exercise capacity in patients with CAD, but only water training was associated with increased levels of NO metabolites after the intervention period – indirectly suggesting an improvement in endothelial function with aquatic exercise. Given the specific hemodynamic responses to exercising in xiphoid-level water, with increased peripheral blood flow and enhanced shear stress yielding endothelial nitric oxide synthase up-regulation (Di Francescomarino et al., 2009; Ayme et al., 2014), improvements in endothelium-dependent vascular function may be hypothesized, and have indeed been confirmed with aquatic exercise in prehypertensive adults (Nualnim et al., 2012) and in patients with osteoarthritis (Alkatan et al., 2016), but not in patients with CAD. Thus, while several studies confirmed the relative cardiovascular safety of thermo-neutral water immersion in patients with CAD, only a limited number of trials assessed the efficacy of aquatic training modalities on aerobic exercise capacity, and none appraised the effect of such training on vascular function or explored its interplay with low-grade inflammation and hemostasis.

Therefore, we sought to compare the effect of a land- and a water-based exercise training program on aerobic exercise capacity and vascular function. In addition, we assumed that the improvements in endothelium-dependent vascular function would be accompanied by a reduction in markers of low-grade inflammation, endothelial adhesion and coagulation, given the association between inflammation and endothelial dysfunction, and the central role of endothelial integrity in promoting cell adhesion and coagulation. Hence, we hypothesized that a 2-week, twice-daily aquatic endurance *plus* calisthenics exercise training program would (i) increase aerobic exercise capacity (peak oxygen uptake/$\dot{V}$O_2_peak), (ii) improve endothelium-dependent flow-mediated vasodilation (FMD), and (iii) reduce markers of low-grade inflammation, cell adhesion, and hemostasis as compared to land-based endurance *plus* calisthenics exercise training and to no exercise in patients undergoing short-term residential cardiac rehabilitation after a recent CAD event.

## Materials and Methods

### Study Design, Setting, and Patients

**FIGURE 1:**
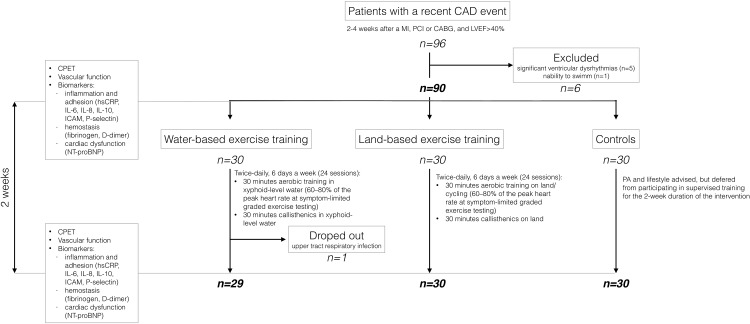
Patient selection and study design flowchart.

The study was designed as a prospective, randomized, open-label clinical trial with three parallel groups (two intervention groups and one control group) (Figure 1).

The study was carried out at the Centre for Cardiac Rehabilitation in Šmarješke Toplice, Slovenia. The center provides residential cardiac rehabilitation for patients after a myocardial infarction or open-heart surgery, with a live-in 14-day program encompassing twice-daily supervised exercise training sessions, education, dietary and smoking cessation advice, medical supervision, and psychological counseling for individuals without access to outpatient rehabilitation services.

Patients after a recent CAD event [2–4 weeks after myocardial infarction, percutaneous coronary intervention (PCI) and/or coronary artery by-pass surgery (CABG)] with a left ventricular ejection fraction (LVEF) above 40% were invited to participate. Recruitment took place between May and October 2016. Patients with uncontrolled/decompensated valve diseases necessitating specific (surgical or percutaneous) management, patients after valve replacement, with uncontrolled dysrhythmias or presence of a permanent pacemaker, with contraindications to exercise, unable to perform exercise testing or to swim, with mental impairment, severe anemia, severe obstructive/restrictive lung disease, recent thromboembolic events, hepatic dysfunction, and/or age over 80 years were excluded.

Patients were randomized into (a) a water-based exercise training group, (b) a land-based exercise training group, or (c) a control group, using adaptive urn-randomization with sealed envelopes and allocation concealment from the recruiting investigator.

Before and after the intervention period (on Day 0 and Day 14), all participants underwent cardiopulmonary exercise testing, ultrasonographic assessment of FMD of the brachial artery, and blood sample collection. The primary outcomes were change from baseline of $\dot{V}$O_2_peak and FMD. Exploratory outcomes included change from baseline of biomarkers of low-grade inflammation (hsCRP, IL6, IL8, IL10) and endothelial activation (ICAM, P-selectin), hemostasis (D-dimer, fibrinogen), and neurohormonal activity (NT-proBNP).

Written informed consent was obtained for each participant. The study complied with the World Medical Association Declaration of Helsinki on ethics in medical research and was approved by a local medical research ethics committee (0120-655/2016-2). This study is registered at ClinicalTrials.gov, number NCT02831829.

### Intervention

The intervention was designed as either a water- or a land-based endurance *plus* calisthenics exercise program during 2-week residential cardiac rehabilitation. The exercise programs consisted of 30-min training sessions twice daily, 6 days a week (24 sessions in total).

Other aspects of rehabilitation (including lifestyle education and provision of a Mediterranean-style diet, medical supervision, psychological support) were identical for both intervention groups. The principles of treatment were not changed over the intervention period, but medication adjustment was allowed at the discretion of the treating cardiologist to ensure optimal control of risk factors.

**Water-based exercise training** comprised two daily training sessions in a heated swimming pool (32.8°C), with water depth at the xiphoid process level (1.5 m). The exercise program consisted of two 30-min sessions daily, namely aerobic endurance and calisthenics. *Aerobic endurance exercise* comprised 5 min of warm-up, 20 min of conditioning (water walking, side-stepping, cycling with arms) at 60–80% peak heart rate achieved during symptom limited graded exercise testing, and 5 min of cool-down. *Calisthenics* comprised 5 min of warm-up, 20 min of conditioning (engaging muscle groups of the upper and lower limbs, such as triceps extensions, triceps dips, modified leg press, leg abduction/adduction, wall push-ups at 60–80% peak heart rate), and 5 min of *cool-down.*

**Land-based exercise training** comprised two 30-min sessions daily, namely bicycle ergometer training (5 min warm-up, 20 min at 60–80% peak heart rate, and 5 min of *cool-down*) and calisthenics (5 min of *warm-up*, 20 min of exercises engaging muscle groups of the upper and lower limbs at 60–80% peak heart rate with a progressive increase in speed and the number of repetitions), and *cool-down*.

**Patients in the control group** were given lifestyle advice, and made aware of the beneficial effects of exercise and advised to engage in regular physical activities (i.e., usual daily activities, such as walking), but were asked to refrain from enrolling in a supervised exercise program for the duration of the intervention period (i.e., 2 weeks).

### Assessment of Aerobic Exercise Capacity

Aerobic exercise capacity was determined by measuring $\dot{V}$O_2_peak by cardiopulmonary bicycle exercise testing (CPET) using the cycle-ergometer Schiller CS-200 (Schiller A.G. Baar, Switzerland) with the Ganshorn Power Cube gas analysis unit (Ganshorn Deutschland GmbH). Calibration of primary sensors for flow, O_2_ and CO_2_ gas measurement were performed before each exercise test. All the participants underwent a symptom-limited exercise test. They were advised to adhere to normal medical regimes, avoid exercise and heavy meals on the day of testing. Resting data including ECG were monitored 3 min before starting the test. Participants were tested using a maximal incremental protocol: after 3 min of unloaded cycling (“0 W”), the work rate was continuously increased on the computer-controlled cycle ergometer in a ramp-like fashion to achieve the predicted maximal workload after 10 min. Predicted maximal workload on the bicycle ergometer was estimated based on age, gender, and body surface area. The test was considered completed if the respiratory exchange ratio achieved was ≥1.1. During exercise, participants wore a mouthpiece connected with the gas analysis unit, thus measuring oxygen and carbon dioxide flow ($\dot{V}$O_2_ and $\dot{V}$CO_2_, respectively). ECG and heart rate were continuously monitored, and records were made every 2 min. Blood pressure was measured at rest and every 2 min during the test and cool-down period. Monitoring of the participants and the mentioned parameters continued for 6 min after test termination. There were no clinically relevant adverse effects during the exercise testing. To assess the reproducibility of exercise testing, 10 subjects were selected randomly and tested twice before the intervention started. The intra-class correlation coefficient (ICC for single measure) for $\dot{V}$O_2_peak was 0.861, *p* = 0.004.

### Assessment of Endothelial Function

Endothelial function was assessed by flow-mediated dilatation (FMD) of the right brachial artery with ultrasound scanning (Philips ultrasound system iE 33 with a high resolution linear-array vascular probe with a frequency of 10 MHz), under standardized conditions and in accordance with current recommendations (Flammer et al., 2012). The brachial artery was imaged 2–10 cm above the elbow fossa. To determine the endothelium-dependent vasodilatation, the forearm was tightened with the sphygmomanometer cuff until a pressure of 50 mmHg higher than the systolic pressure value was achieved. The grip was released after 4.5 min. Flow was measured within 15–20 s, and artery diameter 60–90 s after releasing the grip. After 15 min of rest, endothelium-independent vasodilatation was measured, induced by 0.4 mg of nitroglycerin (Nitrolingual spray^®^) upon sublingual spray application. The diameter of the brachial artery and the average velocity of blood flow were measured 3–4 min after dosing. FMD was expressed as percentage change from rest [(brachial artery diameter at peak hyperemia – diameter at rest) × 100/diameter at rest]. To assess the reproducibility of FMD, 10 subjects were selected randomly. The intra-class correlation coefficient for FMD was 0.855, *p* = 0.004.

### Blood Markers

All patients had venous blood samples taken in the fasting state, in the morning, after 30 min of rest in the supine position, from the cubital vein into 4.5 mL vacuum tubes containing 0.11 mol/L sodium citrate (Becton Dickinson, Vacutainer System Europe, Germany). Plasma was prepared within 30 min with 20-min centrifugation at 2,000 × *g*. In fresh plasma, the concentrations of fibrinogen (Dade^®^ Thrombin Reagent) and D-dimer (Innovance D-dimer, both Siemens Healthcare Diagnostics, Marburg, Germany) were determined on an automated coagulation analyzer CS2100i (Sysmex, Kobe, Japan). The remaining plasma was aliquoted, snap frozen in liquid nitrogen and stored at –75°C until analysis. In thawed plasma, NT-proBNP was determined on a Stratus^®^ CS Acute Care^TM^ analyzer based upon solid phase Radial Partition Immunoassay (RPIA) technology (Siemens Healthcare Diagnostics, Marburg, Germany). Plasma CRP, ICAM-1, IL-6, IL-8, IL-10, and P-selectin were measured with the xMAP^®^ Technology utilizing magnetic beads coupled with specific antibodies (all R&D Systems, Minneapolis, United States) on a MagPix instrument (Luminex Corporation, Austin, United States).

### Statistical Analysis

Data are presented as mean (standard deviation) for normally distributed continuous variables and as median (interquartile range) otherwise. Differences in the baseline characteristics of patients between groups were tested by ANOVA or Kruskal–Wallis test, as appropriate. Differences in the primary objective, end-of-study VO_2_ max, between study groups were tested using ANCOVA, controlling for baseline VO_2_ max and age of patients. ANCOVA with age and baseline measurement included as a covariate was used for all other normally distributed variables. *Post hoc* differences were tested by Sidak test. Variables, measured in percentages (FMD and NMD) were logarithmized prior the analysis. For the logarithmized FMD, the assumption of homogeneity of regression slopes was violated, therefore, between and within group ANOVA with age as a covariate was used to test the between-group differences. In all other non-normally distributed variables, the change from baseline for each patient was calculated and Kruskal–Wallis test was used to test the differences in change from baseline between the study groups. When statistically significant differences between groups were found, Mann–Whitney *U* test was used to test pairwise differences. The differences with *p* < 0.05 were treated as statistically significant. All analyses were done using the software SPSS, v. 21.

Sample size calculation suggests an 80% power at 0.05 significance level for the detection of a between-group difference (and assumed between-subject standard deviation) of 1 MET (3.5 ml kg^-1^ min^-1^) with the inclusion of 90 patients (30 per group).

The differences with *p* < 0.05 were treated as statistically significant. All analyses were done using the software SPSS, v. 21.

## Results

### Baseline Characteristics

Eighty-nine patients completed the study: 30 patients in the land-based training group, 29 in the water-based training group, and 30 in the control group; one participant dropped out during the course of the study due to upper respiratory tract infection (Figure 1). Mean age of the participants who completed the study was 59.9 ± 8.2, and 77.5% were male. All the participants had CAD, and 77 (86.5%) had suffered a myocardial infarction. Patients randomized to the land-based group were significantly older and achieved a lower $\dot{V}$O_2_ peak at baseline (Table 1).

**Table 1:** Baseline characteristic of the patients who completed the study.

Variables	Patients who completed the study (*n* = 89)	Land-exercise group (I) (*n* = 30; 33.7%)	Water-exercise group (II) (*n* = 29; 32.6%)	Control group (III) (*n* = 30; 33.7%)	*P*^∗^
Age Mean(SD), years	59.9 (8.2)	62.4 (7.6)	56.7 (8.4)	60.6 (8.3)	0.026 (I–II)
Gender m/f, *n* (%)	69 (77.5)/20 (2.5)	21 (70)/9 (30)	24 (82.8)/5 (17.2)	24 (80)/6 (20)	0.464
					0.523
MI + PCI *n* (%)	60 (67.4)	19 (63.3)	17 (58.6)	24 (80)	
PCI *n* (%)	3 (3.4)	1 (3.3)	2 (6.9)	0 (0)	
CABG *n* (%)	9 (10.1)	3 (10)	3 (10.3)	3 (10)	
MI + CABG *n* (%)	17 (19.1)	7 (23.3)	7 (24.1)	3 (19)	
HBP *n* (%)	52 (58.4)	18 (60)	14 (48.3)	20 (66.7)	0.350
Dyslipidemia *n* (%)	65 (73)	19 (63.3)	22 (75.9)	24 (80)	0.318
Family history *n* (%)	53 (59.6)	19 (63.3)	18 (62.1)	16 (53.3)	0.692
Obesity *n* (%)	20 (22.5)	6 (20)	7 (24.1)	7 (23.3)	0.921
DM *n* (%)	15 (16.9)	3 (10)	5 (17.2)	7 (23.3)	0.385
Low physical activity level *n* (%)	44 (49.4)	17 (56.7)	15 (51.7)	12 (40)	0.415
Smoking status *n* (%)	48(53.9)	15 (59)	16 (55.2)	17 (53.9)	0.863
Aspirin *n* (%)	86 (96.6)	29 (96.7)	29 (100)	28 (93.3)	0.366
β blockers *n* (%)	76 (85.4)	26 (86.7)	24 (82.8)	26 (86.7)	0.887
Statins *n* (%)	79 (88.8)	26 (86.7)	26 (89.7)	27 (90)	0.906
ACE/ARB *n* (%)	72 (80.9)	24 (80)	22 (75.9)	26 (86.7)	0.566


*SD, standard deviation; IQR, interquartile range; CI, confidence interval; CAD, coronary artery disease; MI, myocardial infarction; PCI, percutaneous coronary intervention; CABG, coronary artery by-pass graft; HBP, high blood pressure; DM, diabetes mellitus; ACE, angiotensin-converting enzyme inhibitor; ARB, angiotensin II receptor blockers. ^∗^Annotations for groups that differ as found by *post hoc* tests are provided in the brackets.*

### Aerobic Exercise Capacity

**FIGURE 2:**
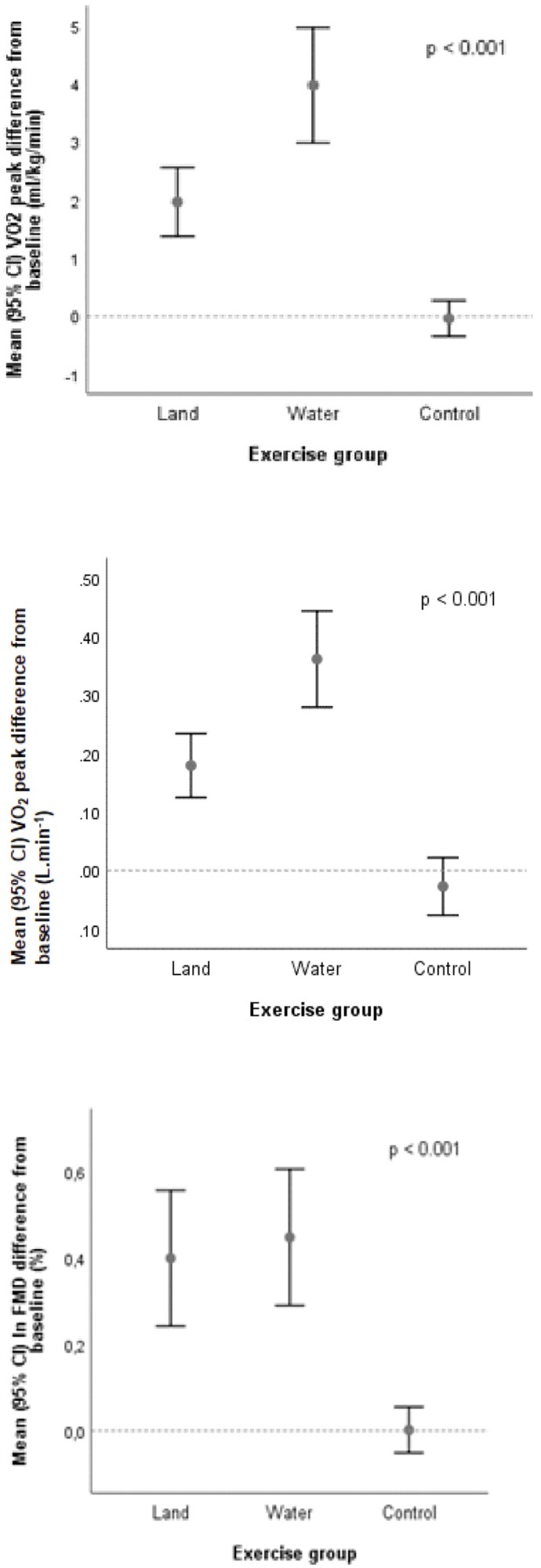
Changes in aerobic exercise capacity ($\dot{V}$O_2_peak) and flow-mediated dilatation (FMD) expressed on the logarithmic scale.

Both exercise modalities were associated with a statistically significant increase in $\dot{V}$O_2_peak as compared with controls (Figure 2). After controlling for baseline $\dot{V}$O_2_peak and patients’ age (ANCOVA), mean estimate end-of-study $\dot{V}$O_2_peak increased by 15.3% (16.7 ml kg^-1^ min^-1^; 95% CI 16.0–17.4 ml kg^-1^ min^-1^; *p* < 0.001 for change from baseline) with land-based training, and by 27.4% (18.6 ml kg^-1^ min^-1^; 95% CI 17.9–19.3 ml kg^-1^ min^-1^; *p* < 0.001 for change from baseline) with water-based training, but not in controls (a 0.6% increase, i.e., 14.9 ml kg^-1^ min^-1^, 95% CI 14.2–15.6; 14.9 ml kg^-1^ min^-1^; *p* = 0.775 for change from baseline). The effect size (*d*) was moderate in the land-based group (*d* = 0.61), and large in the water-based group (*d* = 1.02). Time-to-exhaustion and peak workload also increased significantly in both intervention groups compared to controls (Table 2).

**Table 2:** Measurements for the three study groups at baseline and after training, and between group differences.

Variables	Land-exercise group (I)				Water-exercise group (II)				Control group (III)				*P*^∗^
				
	Baseline	After	Δ%	*p*	Baseline	After	Δ%	*p*	Baseline	After	Δ%	*p*	
VO_2_ peak ml kg^-1^ min^-1^	13.1 (2.8)	15.1 (3.2)	15.3	0.001	14.6 (3.3)	18.6 (3.9)	27.4	0.001	16.6 (3.6)	16.5 (3.8)	-0.6	0.775	0.001
VO_2_ (L min^-1^)	1.07 (0.27)	1.25 (0.33)	16.8	<0.001	1.31 (0.34)	1.67 (0.41)	27.4	<0.001	1.47 (0.38)	1.45 (0.40)	-1.3	0.265	<0.001 (all)
TTE (sec)	605 (91)	710 (128)	17.3	<0.001	710 (123)	864 (158)	21.6	<0.001	796 (150)	800 (145)	0.5	0.493	<0.001 (all)
RER	1.10 (1.10–1.10)	1.12 (1.1–1.16)	1.8	0.001	1.10 (1.10–1.11)	1.15 (1.10–1.19)	4.5	0.001	1.10 (1.10–1.11)	1.10 (1.10–1.13)	0.0	0.026	0.026 (II vs. III)
Peak workload (Watt)	70 (15)	88 (21)	25.7	<0.001	88 (21)	113 (26)	28.4	<0.001	103 (25)	104 (24)	0.9	0.755	<0.001 (all)
FMD (%)	5.5 (3.1–8.6)	8.8 (5.3–11.4)	37.7	<0.001	7.2(4.0–8.7)	9.2 (7.4–12.9)	44.9	<0.001	7.0 (3.7–8.6)	6.4 (3.7–8.5)	-1.5	0.692	<0.001 (I–III, II–III)
NMD (%)	10.6 (7.0–13.3)	11.1 (9.0–14.3)	11.6	<0.001	10.5 (8.5–14.0)	11.4 (7.6–13.4)	5.4	0.042	10.9 (6.0–13.4)	11.6 (7.4–14.2)	2.9	0.579	0.013 (I–III)
Fibrinogen g L^-1^	3.0 (3.0–3.4)	3.2 (3.0–3.5)	6.7	0.875	3.3 (3.0–3.9)	3.0 (2.7–3.8)	-9.1	0.248	3.0 (2.7–3.4)	3.0 (2.6–3.3)	0.0	0.791	0.565
D dimer μg L^-1^	625 (280–1220)	465 (2701070)	-25.6	0.066	400 (270–810)	370 (260–590)	-7.5	0.001	310 (200–510)	270 (200–380)	-12.9	0.129	0.099
CRP mg/L	0.7 (0.4–1.7)	0.6 (0.4–2.0)	-9.1	0.734	0.9 (0.6–1.8)	0.5 (0.3–1.0)	-50.0	0.133	0.5 (0.4–1.3)	0.5 (0.3–2.0)	10.2	0.829	0.256
IL6 ng L^-1^	10.1 (7.5–11.7)	10.9 (8.5–12.4)	7.9	0.211	9.6 (7.5–11.3)	9.3 (7.2–11.0)	-3.1	0.380	7.5 (6.9–9.6)	7.4 (6.2–9.9)	-1.3	0.041	0.090
IL8 ng L^-1^	19.6 (16.4–21.3)	18.4 (16.4–22.9)	-6.1	0.755	17.8 (16.6–20.7)	19.2 (16.0–20.5)	7.9	0.927	19.5 (16.6–23.0)	19.0 (16.6-20.1)	-2.6	0.034	0.187
IL10 ng L^-1^	17.6 (12.7–21.7)	17.2 (12.7–25.7)	-2.3	0.703	16.8 (12.7–25.7)	15.8 (10.6–21.0)	-6.0	0.710	14.7 (12.7–15.8)	14.2 (11.6–16.7)	-3.4	0.038	0.360
ICAM μg L^-1^	372 (270–506)	362 (275– 567)	-2.7	0.642	523 (362–873)	514 (362–801)	-1.7	0.554	662 (507–806)	667 (520–787)	-0.8	0.325	0.515
Selectin mg L^-1^	27.0 (6.8)	27.9 (7.9)	3.3	0.140	25.4 (4.9)	25.1 (4.9)	-1.2	0.626	27.8 (5.6)	26.9 (5.9)	-4.3	0.101	0.060
NT-pro-BNP ng L^-1^	707 (401–1604)	669 (335–1360)	-5.4	0.074	396 (296–541)	Δ308 (187–394)	-1.2	0.001	111 (66–221)	111 (52–297)	-4.3	0.681	0.007 II–III
BMI kg m^-2^ median (IQR)	29 (26.5–31.6)	28.9 (26.9–30.7)	-0.3	0.104	30 (27.1–32.6)	29.1 (27.0–31.5)	-3.0	0.001	29.3 (26.8–30.9)	29.2 (26.8-32.0)	-0.3	0.144	0.082


*Data are shown as mean and standard deviation (SD) or median and 25th percentile – 75th percentile. TTE, time-to-exhaustion; RER, respiratory exchange ratio; FMD, flow-mediated dilatation; NMD, nitroglycerin-mediated dilatation; CRP, C-reactive protein; IL, interleukin; ICAM, intercellular adhesion molecule; NT-proBNP, N-terminal prohormone B-type natriuretic peptide; BMI, body mass index. *p*^∗^, *p* between groups (ANCOVA).*

### Vascular Function

End-of-study FMD increased from 5.5 to 8.8% (*p* < 0.001) in the land-based training group, and from 7.2 to 9.2% (*p* < 0.001) in the water-based training group; no significant change was observed in the control group (*p* = 0.629). NMD increased in both intervention groups (with larger increments in the land-based exercise group); the increase in the intervention groups was statistically significant in comparison to controls (*p* < 0.001). See Table 2 and Figure 2.

### Biomarkers

No significant changes were detected in biomarkers of low-grade inflammation, whereas levels of NT-proBNP and D-dimer decreased in the water-based training group (*p* = 0.01 for both; Table 2).

## Discussion

Supervised short-term exercise training – either water-based or land-based – is safe, and improves aerobic exercise capacity and vascular function in patients with CAD. Despite concerns about the safety and effectiveness of aquatic exercise in patients after a recent CAD event, our study in patients undergoing residential cardiac rehabilitation demonstrated that a 2-week, twice-daily water-based training program was not associated with adverse cardiovascular events, and improved aerobic exercise capacity (as determined by $\dot{V}$O_2_peak) and endothelial function (as determined by FMD). To our knowledge, this is the largest study of water- vs. land-based training in patients after a recent CAD event, and the first to address vascular function in this context. Our results contribute to the growing body of evidence on the safety and effectiveness of aquatic exercise in cardiovascular patients, and suggest that aquatic exercise modalities may be a suitable option for cardiac rehabilitation of selected patients after a recent CAD event, such as those with concomitant musculoskeletal conditions, frailty or at risk of falls, or for those who might prefer aquatic exercise.

### Aerobic Exercise Capacity

In our study, both land- and water-based training were associated with a significant increase in exercise capacity ($\dot{V}$O_2_peak, time-to-exhaustion, and peak workload) as compared to controls. The magnitude of baseline-adjusted $\dot{V}$O_2_peak increase – in the range of 2–4 ml kg^-1^ min^-1^, roughly corresponding to 1 MET – was comparable in the two intervention groups, and represents a clinically significant achievement (Conraads et al., 2015). $\dot{V}$O_2_peak improvements in our study were larger than those reported in previous randomized trials of short-term (3 weeks) aquatic rehabilitation (in the range of 2.0–2.4 ml kg^-1^ min^-1^) (Laurent et al., 2009; Teffaha et al., 2011). Both Laurent et al. (2009) and Teffaha et al. (2011), however, enrolled more stable and younger CAD patients (mean age 52 and 54 years, respectively), which was reflected in higher baseline $\dot{V}$O_2_peak (20 and 22 ml kg^-1^ min^-1^, respectively) and therefore possibly disposed the study population to diminishing returns in aerobic exercise capacity improvements. In fact, the end-of-study $\dot{V}$O_2_peak in our population (16.7 and 18.4 ml kg^-1^ min^-1^, respectively) was still lower than that reported in studies with CAD patients (Casillas et al., 2007; Winzer et al., 2018), but nonetheless higher when compared to studies of water-based training in patients with chronic heart failure. This likely reflects the specifics of our patient population (i.e., after a recent myocardial infarction and/or revascularization procedure in the risk spectrum between stable CAD at one end, and clinically manifest cardiac dysfunction on the other); of note, our patient population represents the population of CAD patients traditionally referred for cardiac rehabilitation.

End-of-study $\dot{V}$O_2_peak in our trial was also significantly higher in the aquatic exercise group (*p* < 0.001 after co-variance analysis adjusting for age and baseline capacity). Larger improvements with water- vs. land-based exercise training in CAD were observed previously (Teffaha et al., 2011; Lee et al., 2017). However, the magnitude of difference (2 ml kg^-1^ min^-1^) was larger in our study when compared to previous reports (1 ml kg^-1^ min^-1^) (Mourot et al., 2010; Teffaha et al., 2011), but the confidence intervals for our estimations were large and the study was underpowered to provide a definite conclusion as to whether differences in end-of-study $\dot{V}$O_2_peak between aquatic and land-based training are indeed relevant. Methodologically, the difference favoring water-based over land-based exercise in our study may still be attributable to randomization failure (with patients in the land-based group being older and having lower baseline $\dot{V}$O_2_peak). Alternatively, larger improvements may derive from either the specific physiology of water immersion or the higher intensity of aquatic exercise. In terms of specific physiology, water immersion is linked with hemodynamic and peripheral responses associated with improved myocardial efficiency and endurance (DiCarlo et al., 1991; Tei et al., 1995; Mourot et al., 2010). Previous studies (Teffaha et al., 2011; Lee et al., 2017) compared water- vs. land-based calisthenics on top of land-based endurance training (cycling), whereas our aquatic training protocol (both endurance and calisthenics) was entirely carried out in xiphoid-level water. In terms of the intensity, exercise prescription was based on peak heart rate achieved during symptom-limited graded exercise testing on a land bicycle ergometer. On the one hand, the hypothesized intensity may not be directly interchanged between aerobic exercise in water and cycling on ambient air. On the other hand, peak heart rate-derived intensity may not provide a precise measure of metabolic stress in comparison to, for instance, ventilatory thresholds, especially in patients with cardiovascular disease (Carvalho and Mezzani, 2010). The challenges of threshold appraisal in clinical practice, however, partially explain why the majority of research – including ours – continues to favor the use of peak heart rate to prescribe exercise (Mann et al., 2013). Moreover, our intervention protocol did employ the aquatic heart rate adjustment (i.e., to a maximum rate 13% or 10 bpm lower than in land-based exercise) (Gabrielsen et al., 2000). In this respect, previous research yielded inconclusive results: some studies have corroborated the higher intensity (and thus the rationale for heart rate adjustment) of aquatic exercise (Lee et al., 2017), while others – including research in CAD populations exercising to up to 75% $\dot{V}$O_2_peak – have not (Tokmakidis et al., 2008; Laurent et al., 2009; Teffaha et al., 2011). These inconclusive results may be reconciled by addressing the specific protocols of aquatic exercise, suggesting that the level of water may play an important role. In our study, deeper water (xiphoid process level) – which increases buoyancy and decreases resistance (Cider et al., 2003) – may have provided comparable intensity of aquatic exercise, but a significant hydrostatically induced hemodynamic response to immersion.

### Vascular Function

Both water- and land-based exercise training improved vascular function (between 2 and 3 absolute percentage change improvements). Similar improvements have been reported with land-based aerobic training (Di Francescomarino et al., 2009), but not yet with aquatic exercise in patients with CAD. FMD – a marker of endothelial function and thus cardiovascular health (Flammer et al., 2012) – increased significantly after 2 weeks of exercise training in both intervention groups. While most previous studies employed exercise programs of longer duration, our intervention was relatively short-term and suggests that an increase in endothelial function can be detected as soon as 2 weeks after exercise training initiation; these results are in line with previous reports in both human trials (Tinken et al., 2008) and animal models (Graham and Rush, 2004). Improved endothelial function – as determined indirectly by increased levels of plasma nitric oxide metabolites – has been reported in water-based exercise training in patients with CAD (Conraads et al., 2015), while FMD increases have been documented in prehypertensive adults (Nualnim et al., 2012) and patients with osteoarthritis undergoing aquatic exercise training (Alkatan et al., 2016). Contrary to our findings, however, Alkatan et al. (2016) – in the only previous study comparing water-based training (swimming) with land-based training (cycling) – reported on larger improvements in endothelial function with the former (4 vs. 1% absolute percentage change improvement). Exercise type (swimming) and duration (8 weeks) may partly explain such differences; alternatively, Alkatan et al. (2016) enrolled cardiovascular disease-free individuals with osteoarthritis, whereas in our CAD population the vascular damage may have been too pronounced for a discernible difference between water- and land-based training to be detected.

### Inflammation, Neurohormonal Activity, Hemostasis, and Endothelial Activation

Contrary to the increased aerobic exercise capacity and vascular function, neither inflammation nor endothelial activation markers improved. Atherosclerosis in general, and CAD in particular, are characterized by low-grade inflammation, which may be reduced with regular long-term exercise training (Nimmo et al., 2013). We hypothesized that improvements in endothelium-dependent vascular function would be accompanied by a reduction in the markers of low-grade inflammation, endothelial adhesion and coagulation, given the association between inflammation and endothelial dysfunction, and the central role of endothelial integrity in promoting cell adhesions and coagulation. However, 2 weeks of exercise training may have been too short to achieve such changes; aquatic exercise trials of longer duration in osteoarthritis (Alkatan et al., 2016) have reported improved vascular function and inflammation markers with water-based exercise. Adding to these observations, our study suggests that improvements in FMD – unparalleled by a reduction in the markers of inflammation (interleukins and hsCRP) and inflammation-induced endothelial activation (P-selectin and ICAM) – more likely derive from immediate exercise-induced hemodynamic changes rather than from a reversal in inflammation-caused vascular dysfunction. Alternatively, it is also possible that exercise-induced long-term changes in body mass, composition and metabolism might in the long run reverse low-grade chronic inflammation in cardiovascular disease; however, neither our 2-week exercise program nor longer 12-week trials (Mohammadi et al., 2018) achieved significant changes in the body mass index, and did not appraise potential body composition changes, which may be brought about by aquatic exercise.

### Limitations

We have identified several limitations. Firstly, this was a single-center study involving a limited number of patients with a recent CAD event. The results can therefore not be extrapolated to other cardiovascular patients. Secondly, we assessed relevant but surrogate endpoints, and the clinical relevance of our findings should be confirmed in larger clinical trials. Also, our study focused on exercise capacity, vascular function and low-grade inflammation; whilst providing some insight into aquatic exercise in patients with CAD, our findings convey only limited inferences about the potential (patho)physiologic responses to water- vs. land-based exercise training in this patient population. Specific impacts of aquatic exercise in patients with CAD – such as on body composition and metabolism – should therefore be addressed in further studies. Thirdly, baseline between-group differences suggest randomization failure and have required statistical adjustment, which calls for our study to be regarded as pilot and hypothesis-generating. Fourthly, while both intervention groups underwent residential cardiac rehabilitation (controlling for some confounders, such as diet), the control group did not, which may have yielded overestimation of the effect of both interventions as compared to controls. Lastly, we chose a specific type (xiphoid-level endurance *plus* calisthenics training) and duration of exercise (2-week intervention), which can only address immediate physiological responses, but not sustainable effects of regular training.

## Conclusion

Aquatic exercise is a safe and effective training modality for patients undergoing short-term residential cardiac rehabilitation after a recent CAD event. As compared to land-based exercise, endurance *plus* calisthenics exercise training in thermo-neutral water provides comparable improvements in exercise capacity and vascular function in patients with CAD. Our pilot study therefore represents a starting point for further research into optimal exercise modalities in CAD patients, with water-based training likely emerging as a suitable exercise option.

## Ethics Statement

Patients who corresponded to the inclusion criteria were invited to participate in the study. A written informed consent was obtained for each participant. The study complied with the World Medical Association Declaration of Helsinki on ethics in medical research and was approved by the local medical research ethics committee (0120-655/2016-2).

## Author Contributions

DV contributed to drafting the work, acqusition, analysis, and interpretation of the data for the work, and agreed to be accountable for all aspects of the work in ensuring that questions related to the accuracy or integrity of any part of the work are appropriately investigated and resolved. MN and MB substantially contributed to the conception of the work. BB substantially contributed to the design of the work. BJ contributed to the drafting the work, revising it critically for important intellectual content, and provided approval for the final manuscript.

## Conflict of Interest Statement

The authors declare that the research was conducted in the absence of any commercial or financial relationships that could be construed as a potential conflict of interest.
